# 

**Published:** 1910-04

**Authors:** 


### EDITORIAL DEPARTMENT.

The Annual Meeting of the Texas State Medical Association will be held at Dallas May 10, 11, 12 prox. It will be a rouser. A splendid program has been prepared, science and social, and all are anticipating a royal good time. Those Dallas people know how to entertain and always "set 'em up" for the doctors in *de luxe* style. But it will not be all frolic. There are serious and important matters to be settled, an indication of which we gave in last issue. There will be some lively tilts I have no doubt over some of the proposed reforms, and yours truly will surely make one more determined stand for what he believes to be the true and best interests of regular, legitimate, scientific medicine, the rights of the rank and file, emancipation from boss rule and tyranny of the House of Delegates and restoration of the former status of domination by the true aristocrats of medicine, the "old school," and equal rights to all and special privileges to none. Every member is entitled to a vote on all matters pertaining to the interests of the profession, and all are entitled to have their papers published. *Aut Cæsar aut nullus!*

